# Psychosocial Factors Associated With Resilience Among Iranian Nurses During COVID-19 Outbreak

**DOI:** 10.3389/fpubh.2021.714971

**Published:** 2021-08-04

**Authors:** Davood Afshari, Maryam Nourollahi-darabad, Niloofar Chinisaz

**Affiliations:** Department of Occupational Health Engineering, School of Public Health, Ahvaz Jundishapur University of Medical Sciences, Ahvaz, Iran

**Keywords:** psychosocial, stress, nurses' resilience, COVID-19, healthcare workers

## Abstract

**Background:** In the face of COVID-19, healthcare workers need to cope with the ongoing stressors at play and keep psychological distress at a minimum level. This study examined the psychosocial and demographic factors associated with nurse's resilience in the hospitals of Ahvaz that is one of the top cities infected with COVID-19 in Iran.

**Methods:** The present cross-sectional study was conducted on 387 Iranian nurses in Ahvaz city. For data collection purposes, three online questionnaires (including Copenhagen Psychosocial, Demographic, and Connor–Davidson Resilience Scale) were distributed among the participants.

**Results:** The mean resilience score was equal to 61.8 ± 14.8 for 387 nurses. Resilience had a statistically significant negative correlation with quantitative demand (*r* = −0.273, *P* < 0.008), work pace (*r* = −0.262, *P* < 0.011), emotional demand (*r* = −0.226, *P* < 0.030), stress (*r* = −0.458, *P* < 0.000), and burnout (*r* = −0.287, *P* < 0.005). Multiple linear regression analysis indicated that stress, job satisfaction, burnout and age were the main predictors of nurses' resilience during the (COVID-19) pandemic (*R*^2^ = 0.45).

**Conclusions:** We identified psychosocial and demographic predictive factors that may contribute to greater resilience among nurses during the COVID-19 outbreak. The findings of this study can be used to implement psychosocial interventions to amplify the resilience of medical staff during the COVID-19 outbreak.

## Introduction

Coronavirus disease (COVID-19) was first reported in Wuhan, China, in late December 2019 (1). The new coronavirus, the agent creating COVID-19 disease, is mostly transmitted via respiratory droplets by patients. Occupational exposure is one way through which the disease may be contracted and, thereby, many people are at risk of catching this disease due to their job nature. In this respect, nurses are at the frontline of controlling and treating this disease. Since this disease is highly contagious, nurses are more exposed to the risk of this disease. Hence, it can be argued that nursing is one of the high-risk occupations during the COVID-19 pandemic (2). According to recent studies, healthcare workers are more than 7-fold higher at risk of severe COVID-19 and be vectors of transmission.

In Iran, the first definitive disease infection case was reported on February 19, 2020. Within 10 days from the report of the first COVID-19 death case in Iran, this disease spread to 19 provinces out of the 31 provinces of the country (3). The epidemiological studies conducted from January 30 to March 27, 2020, one of the hospitals in Tehran showed that 12,870 patients have been referred to the emergency ward of the hospital, out of whom 2,968 have been hospitalized with the diagnosis of COVID-19 (4). In a short time after being announced pandemic, many provinces in Iran reported healthcare services saturation because of severe care needs. In many provinces, the high rate of prevalence and mortality for the disease created a serious crisis (5).

The process of spreading this disease has been on the rise in Iran since its inception. Due to the lack of healthcare workers (especially nurses), personal protective equipment (PPE) and the prolonged use of PPE, high rates of physical and mental workload have been reported among the medical personnel during the COVID-19 pandemic (6). In addition to the high physical workload of healthcare workers, psychosocial risk factors, including a wide range of variables can influence their health, safety, and welfare. Such factors are widely in interaction with each other at the individual, collective, and organizational levels in a multidimensional manner. Therefore, considering the current situation resulting from this pandemic, healthcare workers are exposed to depression, anxiety, insomnia, and distress. In this regard, several cross-sectional studies have reported the prevalence of depression and anxiety symptoms among Chinese people during the COVID-19 pandemic (7–9).

In the face of COVID-19, one needs to cope with the ongoing stressors at play and keep psychological distress at a minimum level. When nurses work in such critical conditions and also experience high levels of stress and fatigue, resilience is the key factor that can help them cope with these conditions (10). Resilience assists people to return to the previous state or adapt successfully despite the existence of adverse conditions. This concept has been referred to as a multidimensional feature that helps a person successfully tackle the challenges ahead and enhances protection against stressful situations (11). Thus, the dominance of resilience among healthcare workers is important because measures of greater resilience have previously been connected to the lower levels of psychological and emotional distress and reduced adverse effects of stress on health-related life quality in different groups (12–15). Previous research findings strongly support the importance of resilience in the quality of working life in nurses (12); however, little is known about the factors associated with resilience in this population, especially during the COVID-19 pandemic. When different groups of employees and workers have information about the factors associated with resilience, they may know how to identify the possible methods of resilience promotion. This can be at play especially in the case of healthcare workers during the COVID-19 pandemic. According to psychologists, factors such as personality traits, family ties, and social systems amplify resilience (10).

This study attempts to provide nurses with a better perception of mental health by gathering data on resilience. To this end, a survey involving self–administered questionnaires was conducted to recognize a range of likely demographic and psychosocial factors associated with the higher or lower levels of resilience during the COVID-19 pandemic. The results of this study are expected to contribute to the identification of effective factors in enhancing resilience level among nurses and to the selection of appropriate strategies to manage the stress of dealing with the COVID-19 pandemic by hospital managers.

## Method

### Participants

In the present cross-sectional study, nurses who worked in hospitals affiliated to the University of Medical Sciences, Khuzestan province, Iran, were invited to participate in the research process. The qualified nurses were selected by census sampling from hospitals, from April to May 2020. There are 18 hospitals in the city of Ahvaz, of which, nine hospitals are affiliated to the university of Ahvaz medical science. Of these nine hospitals, four hospitals were designated as referral centers for patients with COVID-19.

The eligible nurses were selected by census sampling based on the inclusion criteria. The inclusion criteria were as follows: (a) having more than 1 year of clinical work experience (b) clinical work experience with patients with COVID-19 for more than 2 months. Since this study has been conducted during the COVID-19 pandemic, the questionnaires were made and sent to nurses online for data collection in order to reduce contact and face-to-face communication. All participating nurses signed a written informed consent form before participation, confirmed by the Ethics Review Committee of Ahvaz Jundishapur University of Medical Sciences (reference number IR.AJUMS.REC.1399.704).

### Survey Measures

#### Demographic Questionnaire

The participants provided detailed information on a range of demographic items, including their age, gender, marital status, number of children, work experience, and education.

#### Connor–Davidson Resilience Scale (CD-RISC)

Resilience was assessed using the Connor–Davidson Resilience Scale (CD-RISC). CD-RISC Scale contains twenty-five items that are graded based on a Likert scale from not true at all (0) to true nearly all of the time (4). Thus, the questionnaire score ranges from 0 (lower degree of resilience) to 100 (greater degree of resilience). Resilience is determined by a CD-RISC score higher than 80. In this regard, Connor and Davidson have documented the Cronbach's alpha coefficient of the Resilience Scale to be 0.89. Moreover, for this scale, the reliability coefficient of 0.87 has been reported (16, 17). The reliability of this scale in Iran has also been assessed and the Cronbach's alpha coefficient of 0.89 has been reported for it (18).

#### Copenhagen Psychosocial Questionnaire (COPSOQ)

The second version of the Copenhagen Psychosocial Questionnaire was used to evaluate the psychosocial risk factors in this study. This questionnaire is one of the most comprehensive standard questionnaires that cover a wide range of psychological factors. Each scale is analyzed within the range of 0 to 100 where zero indicates the minimum degree of risk and 100 represents the maximum risk. The respondents to the COPSOQ are asked to respond to the questions using items on a 5-point scale, most of which are as A = Always, B = Often, C = Some, times, D = Seldom, and E = Never/Hardly or as A = To a very large extent, B = To a large extent, C = Somewhat, D = To a small extent, and E = To a very small extent. Based on the subjects' ratings, each item (scores A to E) scored 0, 25, 75, and 100, respectively, and the average score of the items on each of the factors determined the score of that factor. A lower rating for each of the factors indicates better and ergonomic psychosocial conditions of the work environment (19, 20). The reliability and validity of the Persian version of this questionnaire have been determined through Cronbach's alpha and the values of 0.75 and 0.89 have been reported for them (21).

### Statistical Analyses

Kolmogorov–Smirnov statistical test was run to examine the normality of data distribution. The results showed that all data had a normal distribution; thus, differences in resilience scores between demographic characteristics with healthcare workers were assessed using the independent-samples t- test and one-way ANOVA. Pearson correlation test was also used to examine the associations of resilience with demographic and psychosocial variables. Then, the factors influencing resilience were carried out using multiple linear regression.

## Results

### Demographic Information

Out of the 699 questionnaires sent online, 387 questionnaires were filled out, which is representative of the return rate of 55%. The demographic information of the participants is given in Table 1.

**Table 1:** Demographic information of the study participants (*N* = 387).

**Variable**	**Mean (SD)**
Age (years)	34.42 ± 8.35
Work experience (years)	8.31 ± 3.74
Gender	*n* (%)
Female	236 (61)
Male	151 (39)
Marital status	*n* (%)
Single	199 (51.4)
Married	188 (48.6)
Education level	*n* (%)
Associate degree	47 (12.1)
Bachelor degree	198 (51.2)
Master degree	142 (36.7)
Parent status	*n* (%)
Childless	168 (43.4)
With child	219 (56.6)

### Psychosocial Factors

Psychosocial factors were investigated using COPSOQ. The results showed that quantitative demand, emotional demand, quality of leadership, work-family conflict, burnout, stress, and job satisfaction were the psychosocial factors with high scores. It was also revealed that the lowest mean score belonged to the role clarity (Table 2).

**Table 2:** Mean and Standard deviations of COPSOQ dimensions (*N* = 387).

**Dimension**	**Mean**	**± SD**
Quantitative demand	62.3	20.7
Work pace	45.2	19.4
Emotional demand	65.9	23.5
Influence	39.8	19.8
Possibilities for development	44.7	21.5
Meaning of work	29.8	20.9
Commitment to the workplace	31.6	23.8
Predictability	48.2	21.6
Recognition (reward)	34.4	20.2
Role clarity	28.3	19.1
Quality of leadership	61.5	23.3
Social support from supervisor	44.1	19.9
Job satisfaction	67.6	20.8
Work family conflict	74.9	22.5
Trust regarding management	30.1	18.6
Justice	31.3	29.9
General health	50.2	25.2
Burnout	58.5	19.3
Stress	65.4	25.3

### Resilience

The mean score of 61 was determined for CD-RISC. It was also found that twelve percent of the study population had a high level of resilience (CD-RISC score > 80).

As shown in Table 3, participants with master's degree education had the highest resilience score in the study and there was a significant difference between participants with various levels of education in terms of resilience (*P* = 0.03). Based on the statistical analyses, a significant difference was observed between the female and male subjects with regards to the mean of resilience (*P* = 0.03). In this regard, the women had less resilience than men. Regarding work experience, people with more work experience had higher resilience and the difference in resilience was significant (*P* = 0.01) between various groups of work experience. Statistical analyses also showed that resilience is significantly different in various age groups (*P* = 0.04) and it increases with the increase of age.

**Table 3:** Comparison between demographic variables and the CD-RISC scores (*N* = 387).

**Variable**	**Connor–davidson resilience scale (CD-RISC) score**	***P*-value**
**Age groups (year)**
[Table-fn TN1]23–30	59.54 (15.15)	0.04^**^
[Table-fn TN1]30–37	60.44 (14.71)	
[Table-fn TN1]37–44	63.22 (13.24)	
[Table-fn TN1]44–51	67.35 (14.24)	
**Work experience groups (year)**
[Table-fn TN1]1–5	53.29 (15.1)	0.01^**^
5–10	60.12 (15.26)	
10–15	61.27 (8.3)	
15–20	67.73 (13.4)	
**Gender**
Female	59.53 (14.9)	0.03^*^
Male	62.36 (14.46)	
**Marital status**
Single	62.01 (13.7)	0.34^*^
Married	60.5 (15.73)	
**Education level**
Associate degree	60.02 (12.6)	0.03^**^
Bachelor degree	61.67 (15.3)	
Master degree	63.23 (13.42)	
**Parent status**
Childless	62.339 (13.4)	0.66^*^
With child	60.36 (14.1)	

* *independent t-test.*

** *One-way ANOVA*.

### Factors Associated With Resilience

#### Correlation Analysis

To investigate the correlation of resilience with psychological and demographic variables in the study participants, the Pearson correlation test was run. The results of this analysis are shown in Table 4.

**Table 4:** Correlation between resilience and the psychosocial and demographic factors (*N* = 387).

**Factors**	***r***	***P*-value**
**Demand at work**
Quantitative demand	−0.273	0.008
Work pace	−0.262	0.011
Emotional demand	−0.226	0.030
**Interpersonal relation and leadership**
Quality of leadership	0.219	0.036
**Work individual interface**
Job satisfaction	0.417	0.000
**Health and well-being**
General health	0.301	0.004
Burnout	−0.287	0.005
Stress	−0.458	0.000
**Demographic**
Age	0.304	0.003
Education	0.210	0.044
Work experience	0.226	0.030

The psychosocial factors were significantly correlated with resilience in three domains including, interpersonal relation and leadership, and work individual interface and health and well-being. As shown in Table 4, resilience had a positive correlation with the quality of leadership, job satisfaction, and general health. Therefore, resilience can be enhanced by improving the quality of leadership and job satisfaction and by reducing the quantitative and emotional demands of the job.

As shown in Table 4, those dimensions that had a statistically significant negative correlation with resilience included quantitative demand, work pace, emotional demand, stress, and burnout. This means that resilience is reduced with the increase of quantitative demand, work pace, emotional demand, stress, and burnout.

The results of correlation analysis demonstrated that age, an education level, and work experience were the demographic factors that had a statistically positive correlation with resilience.

#### Multiple Linear Regression Analysis

In order to investigate factors affecting resilience, a multiple linear regression analysis was run. The results of this test showed that stress (β = −0.528, *P* < 0.000), job satisfaction (β = 0.234, *P* < 0.004), burnout (β = −0.143, *P* < 0.045), and age (β = 0.144, *P* < 0.042) were the psychosocial and demographic factors predicting resilience. According to the coefficient of determination (R^2^), 45% of the resilience could be explained by these variables. The regression model has been presented in Table 5.

**Table 5:** Multiple linear regression model for resilience.

	**B**	**S.E**	**β**	***P*-value**
Coefficient	43.813	7.320		
Stress	−0.244	0.038	−0.528	0.000
Job satisfaction	0.191	0.065	0.234	0.004
Burnout	−0.049	0.028	−0.143	0.045
Age	1.544	0.859	0.144	0.076

*R = 0.671; R^2^ = 0.450; Adjusted R^2^ = 0.425*.

## Discussion

In this study, several demographic and psychosocial factors involved with resilience among nurses during the COVID-19 pandemic were assessed. The most significant finding of this study was that resilience was related to stress, age, job satisfaction, and burnout.

### Demographic Factors and Resilience

The results of this study showed that the mean level of resilience in nurses was 61 during the COVID-19 pandemic, which was lower than that of medical staff who work in Radiology Departments (22). Also, the study carried out by Lin et al. on non-native nurses in Wuhan showed that the mean and standard deviation of nurses' resilience in COVID-19 pandemic conditions were equal to 64.86 ± 13.46 (23).

The present study indicated that the nurses' resilience level during the outbreak of COVID-19 was relatively low. Considering the multidimensional nature of resilience, it seems that various factors, including different work environment conditions and existing psychosocial risk factors due to the prevalence of COVID-19 disease have influenced the mean score of nurses' resilience. Therefore, such issues have resulted in a decline in resilience among the nurses.

In terms of resilience in groups with various work experiences, age groups, and education levels, the current findings showed that resilience has experienced an increase with the increase of work experience, age, and education level. Resilience had a significant positive correlation with age, the level of education, and work experience. In this regard, Gillespie et al. assessed the degree of resilience among operating room nurses and reported that nurses' resilience increases with the increase in their experience and education level (24). Similarly, Ang et al. also showed that age and education degree have a significant correlation with resilience. There was a strong association between the highest educational degree and resilience level, and nurses with a bachelor or master's degree had moderate/high resilience three times as much as nurses with only a general nursing certificate (25). Similarly, the results of the study done by Hsieh et al. also maintained that education level is significantly correlated with resilience (26).

The increase of nurses' age, education, and work experience may lead to the progress of their skills with exposure to stress and the development of their abilities to cope with stressful and critical conditions. The progress of such skills helps with the development of different coping strategies, which can simplify their adaptation and provide them with the facility to act usefully and more resiliently in such conditions. Therefore, for increasing resilience in the medical personnel with a lower level of work experience and education, it is strongly recommended to provide them with the relevant training that can enhance their science and experiences in COVID-19 management and coping.

A previous study showed that resilience in female personnel was significantly lower than that in male personnel. The study carried out by Dai et al. showed that a significant difference between male and female medical personnel in terms of the degree of concern about the development of infection among their family members during the COVID-19 pandemic (27). The reason may be attributable to the natural differences between the males and females in their perspectives and methods of looking at critical situations. Women are more sensitive and their anti-stress capability is also relatively weak and, thereby, they suffer from a sense of insufficiency for psychological compatibility. Therefore, more attention and psychosocial support should be assigned to female nurses.

The groups with different marital statuses were not significantly different in terms of resilience. However, the nurses without any children had a higher level of resilience. In fact, the sense of responsibility to the family members is believed to play a significant role in decreasing nurses' resilience during the COVID-19 pandemic. This finding is similar to the results of a study reported by Hsieh et al. (26). The results of a study by Guo et al. also showed that marital status was not associated with resilience (14).

### Psychosocial Factors Associated With Resilience

The most important psychosocial risk factors identified in the current study included work-family conflict, stress, emotional needs, burnout, job satisfaction, and the quality of leadership. In the same vein, Fathi et al. also showed that emotional needs, stress, depression, and anxiety are among the challenges that the healthcare workers might face during the COVID-19 pandemic (28). The results of a similar study conducted by Freimann T & Merisalu demonstrated that work-related psychosocial factors, including quantitative demands, emotional needs, work speed, and role conflict are related to nurses' mental health and these psychosocial factors can produce stress and job burnout among nurses (29). In addition, the results of this study showed that quantitative demand, work pace, emotional demand, stress, and burnout were negatively correlated with resilience in such a way that the increase in the identified negative factors would cause a reduction in resilience among individuals.

Hernandez et al. assessed resilience among medical technicians and reported that resilience has a negative correlation with stress (30). In this regard, prior studies have found that stress, burnout, and fatigue are negatively associated with resilience (11, 31–33). Thus, to increase resilience among nurses, one needs to consider these psychological factors, especially when being involved in a stressful situation arising from unknown diseases.

Work-family conflict was recognized as one of the most important psychosocial risk factors in the present study. In this line, the results of a qualitative study on healthcare workers in Iran showed that conflict with family members is one of the important psychosocial factors for healthcare workers in the COVID-19 pandemic where the majority of families have opposed the presence of healthcare workers at their workplace (28). The results of other studies on psychological factors in the H1N1 pandemic have also suggested that the greatest concern of the healthcare workers was the possible infection of their family members and friends and the health consequences of the disease. This process has led to work-family conflict (34, 35). Thus, training the families and provision of social support in critical conditions can moderate work-family conflict as a psychosocial risk factor affecting resilience.

Another important result of this study was the significant and positive correlation between the quality of leadership and resilience in nurses. The managers of hospitals and wards can exert an important role in providing the necessary conditions for nurses to improve the level of resilience. For example, hospital administrators could implement a training intervention that provides medical personnel with information about the condition of the pandemic exposure and prevention of infectious diseases. These measures may decrease the harmful stress-related effects of exposure to covid-19, thereby, improving their resilience.

A large number of patients, long working hours, and the definition of new roles for nurses in the COVID-19 pandemic conditions lead to an increase in the quantitative and qualitative demands at work that require accurate management and social support. Wang et al. have also emphasized that social supports are related to nurses' resilience (36). Thus, understanding the importance of this issue by managers and improving the quality of management and social support help with the improvement of nurses' resilience. The results of the current study showed that job satisfaction and general health were positively correlated with resilience. Similarly, other related studies have indicated the presence of a significant positive relationship between job satisfaction and resilience (37–39).

In the present study, the results of regression analysis on psychosocial and demographic factors showed that job satisfaction, stress, job burnout, and age have an important role in predicting nurses' resilience level in the COVID-19 pandemic. Indeed, nurses' resilience is negatively associated with job demand, including stress and burnout. These findings emphasize the importance of psychosocial factors in nurse resilience, especially in the COVID-19 pandemic. Therefore, identifying and modifying the factors affecting nurses' resilience in stressful conditions such as pandemics can lead to an increase in resilience. In general, the current findings represent that resilience in nurses can be strongly influenced and maintained by suitable organizational strategies and preventive planning during the prevalence of COVID-19.

### Limitations

In this study, only healthcare workers were assessed. Due to the location and time limitations, this study surveyed only end-point resilience among nurses. Future studies are therefore suggested to employ a longitudinal design study to determine the changes in resilience during the covid-19 pandemic.

## Conclusion

Our paper is the first study that examines the resilience levels of nurses during the outbreak of COVID-19 by exploring associations between resilience and psychosocial factors highlighting important associated risk factors to propose suggestions for amplifying resilience of medical staff during the COVID-19 outbreak. The results of the present study showed that the resilience level of nurses during the outbreak of COVID-19 was low. Thus, the identification of the factors affecting resilience in pandemic conditions and the adoption of related corrective measures are regarded as effective steps to support nurses at the frontline of the fight against this disease. Based on the results of the study, the psychological and demographic predictive factors on resilience, including stress, job satisfaction, burnout, and age should be considered to boost nurses' resilience. In addition, more attention should be paid to other variables that may influence resilience, including education level, work experience, quantitative demand, work pace, emotional demand, quality of leadership, and general health. In general, the findings of this study indicate that the resilience of nurses can be heavily influenced and maintained by the establishment of proper organizational strategies and pre-emptive planning during the outbreak of COVID-19. The information of this study can be used to implement psychosocial interventions to amplify the resilience level of medical staff during the COVID-19 outbreak.

## Data Availability Statement

The original contributions presented in the study are included in the article/supplementary material, further inquiries can be directed to the corresponding author/s.

## Ethics Statement

The studies involving human participants were reviewed and approved by IR.AJUMS.REC.1399.704. The patients/participants provided their written informed consent to participate in this study.

## Author Contributions

All authors made contributions to the study conception and design, made contributions to the interpretation of data. In addition, they also contributed to the drafting and revision of the article as well as approval and, submission of the final version.

## Conflict of Interest

The authors declare that the research was conducted in the absence of any commercial or financial relationships that could be construed as a potential conflict of interest.

## Publisher's Note

All claims expressed in this article are solely those of the authors and do not necessarily represent those of their affiliated organizations, or those of the publisher, the editors and the reviewers. Any product that may be evaluated in this article, or claim that may be made by its manufacturer, is not guaranteed or endorsed by the publisher.
