# Corrigendum: Psychosocial Factors Associated With Resilience Among Iranian Nurses During COVID-19 Outbreak

**DOI:** 10.3389/fpubh.2021.772559

**Published:** 2021-09-30

**Authors:** Maryam Nourollahi-darabad, Davood Afshari, Niloofar Chinisaz

**Affiliations:** Department of Occupational Health Engineering, School of Public Health, Ahvaz Jundishapur University of Medical Sciences, Ahvaz, Iran

**Keywords:** psychosocial, stress, nurses' resilience, COVID-19, healthcare workers

Author Maryam Nourollahi-darabad was accidently place as second author in the published article rather the first author. The updated author list appears below:

Maryam Nourollahi-darabad, Davood Afshari^*^ and Niloofar Chinisaz

The authors apologize for this error and state that this does not change the scientific conclusions of the article in any way. The original article has been updated.

## Publisher's Note

All claims expressed in this article are solely those of the authors and do not necessarily represent those of their affiliated organizations, or those of the publisher, the editors and the reviewers. Any product that may be evaluated in this article, or claim that may be made by its manufacturer, is not guaranteed or endorsed by the publisher.

