# Addendum: Psychosocial factors associated with resilience among Iranian nurses during COVID-19 outbreak

**DOI:** 10.3389/fpubh.2026.1807525

**Published:** 2026-03-09

**Authors:** Davood Afshari, Maryam Nourollahi-darabad, Niloofar Chinisaz

**Affiliations:** Department of Occupational Health Engineering, School of Public Health, Ahvaz Jundishapur University of Medical Sciences, Ahvaz, Iran

**Keywords:** psychosocial, stress, nurses' resilience, COVID-19, healthcare workers

In the published article, clarification is required regarding the relationship between the present manuscript and a previously published article by the authors (1), as well as clarification of certain statistical values reported.

## Transparency statement

Both publications were derived from a single university-approved research project conducted on the same study population. However, the two manuscripts were developed based on distinct objectives, study designs, and analytical frameworks predefined in the approved research protocol.

The article published in Work (1) focused on demographic predictors of nurses' resilience using a general demographic questionnaire. In contrast, the Frontiers in Public Health manuscript aimed to develop a predictive model of nurses' resilience based primarily on psychosocial factors during the COVID-19 pandemic, using the Copenhagen Psychosocial Questionnaire, which includes both psychosocial and demographic components. Accordingly, overlapping demographic variables were reported to ensure transparency, while the primary analytical focus and interpretation differed substantially between the two studies.

## Clarification of statistical discrepancies

Differences observed in reported correlation coefficients and summary statistics between the two publications are attributable to methodological variations in variable extraction, categorization, and reporting.

In the Frontiers manuscript, demographic variables such as age, work experience, and educational level were categorized using more restricted and closed ranges aligned with the Copenhagen Psychosocial Questionnaire and the predictive modeling objectives of the study. In contrast, the Work publication applied broader and more open-ended categories and, for some variables, a higher number of groups.

Because Pearson correlation coefficients are sensitive to variable range, category structure, and sample distribution, these methodological differences sufficiently explain the observed discrepancies in correlation magnitudes and do not indicate the use of different populations or datasets.

## Numerical correction

A minor numerical inconsistency was identified in the abstract of the Work publication (1), where the mean CD-RISC score was reported as 61.18 instead of 61.8. This represents a rounding or reporting inconsistency and does not affect the analyses or conclusions. The correct mean value is 61.8 ± 14.8.
